# CDR3 sequences in IgA nephropathy are shorter and exhibit reduced diversity

**DOI:** 10.1002/2211-5463.13006

**Published:** 2020-11-20

**Authors:** Xi Zhang, Jianming Zeng, Yin Tong, Li Zhang, Xibin Lu, Shenglang Zhu, Zhoufang Li

**Affiliations:** ^1^ School of Life Science and Technology Harbin Institute of Technology Harbin China; ^2^ Core Research Facilities Southern University of Science and Technology Shenzhen China; ^3^ Faculty of Health Sciences University of Macau Macau China; ^4^ Cancer Informatics and System Biology Lab The University of Hong Kong Hong Kong China; ^5^ MD Anderson Cancer Center Houston TX USA; ^6^ Department of Nephrology The 6th Affiliated Hospital of Shenzhen University Health Science Center Shenzhen China

**Keywords:** CDR3 length, IgA, IgA nephropathy, IgG, IgM

## Abstract

Immunoglobulin (Ig) A nephropathy (IgAN) is the most common glomerulonephritis, which is characterized by the deposition of IgA antibody in the glomerulus. Systematic dissection of immune composition may contribute to a better understanding of the alternations in the immune system in IgAN. To this end, here we applied immune repertoire sequencing technology for parallel analysis of the complementary determining region 3 (CDR3) of all B cell receptors, including all five antibody subtypes (*IgA, IgG, IgM, IgE* and *IgD*), in 13 patients with IgAN and 7 healthy individuals. A significant decrease in CDR3 length was observed in the IgAN group. In particular, the JH6 family was significantly increased in IgAN. Amino acid usage was also altered in IgAN. Shannon, Simpson, Gini and Diversity 50 indices also revealed significant differences in the diversity of IgG, IgM and IgA antibodies as compared with controls. The proportions of IgA and IgG were increased, whereas IgM was decreased in IgAN. Moreover, a greater number of CDR3 sequences was shared between patients with IgAN. These findings suggest that the BCR immune repertoire is dramatically altered in IgAN, as characterized by shortened CDR3 length, as well as decreased overall diversity of CDR3.

## Graphical abstract

We report a significant decrease in complementary determining region 3 (CDR3) length in patients with immunoglobulin (Ig) A nephropathy (IgAN). Amino acid usage showed different patterns between patients and healthy control subjects, and the proportions of IgA and IgG were increased, whereas IgM was decreased in patients. These findings suggest that the B cell receptor immune repertoire is altered dramatically in IgAN, as characterized by the shortened CDR3 length, as well as decreased overall diversity of CDR3.

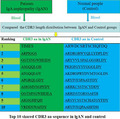

**Abbreviations**
BCR: B cell receptor
CDR3: complementary determining region 3
D50: Diversity 50
Ig: immunoglobulin
IGHV: immunoglobulin heavy‐chain variable region gene
IMGT: international immunogenetics information system
NC: normal control
PBMC: peripheral blood mononuclear cell
RT: reverse transcription
wilcox.test: Wilcoxon rank sum and signed rank tests

Immunoglobulin (Ig) A nephropathy (IgAN) is the most common glomerulonephritis leading to chronic kidney diseases and progressive symptoms. Up to 40% of adult patients with IgAN will experience progressive renal failure and eventually require either dialysis or kidney transplantation within 20 years after diagnostic biopsy [1]. IgAN is characterized by the presence of IgA­dominant or codominant immune complex deposition in the glomeruli. Although IgA is the major Ig component in patients with IgAN, in more than 80% cases, other antibody isoforms, such as IgG, IgM or both, can also be observed in the same region as IgA [2, 3].

Current studies have proposed that several factors may contribute to the IgAN progression. These works studied the genetic mutations that caused structures alteration of IgA protein, the autoantibodies, such as the anti‐glycan IgG and IgAs, as well as external stimulations, such as virus infection, that caused tonsillitis and induced prolonged production of nephritogenic IgA1 [4]. However, none of them alone is sufficient to induce IgAN. First, the genetic factors that can lead to the increased synthesis of abnormal IgA1 with galactose‐deficient O‐glycans are also frequently found in the relatives of patients with IgAN but no signs of renal disease [5, 6]. Second, the anti‐glycan autoantibodies recognize that the O‐glycans in the galactose‐deficient IgA1 in patients with IgAN will form O‐glycan or polymerization of IgAs or IgA–IgG complex [7, 8, 9]. But neither the O‐glycan form nor the polymerization state determines the clearance rate of these abnormal immune complexes in circulation, and they are not necessarily associated with glomerular deposition of IgA in patients with IgAN [10, 11]. Third, infection of the mucosal immune system by extrinsic microorganisms, such as Epstein‐Barr Virus, can cause tonsillitis and induce prolonged production of nephritogenic IgA1 [12, 13]. Similarly, tonsillectomy alone is not enough to increase remission rate in IgAN, but after combining with immunosuppressive steroid treatment at the same time, it can improve the remission rate in a long‐term effect [14, 15, 16, 17]. The scientific rationale for tonsillectomy or tonsillectomy in combination with immunosuppressive therapy for IgAN remains obscure. In summary, none of the genetic factors, the abnormal IgA immune complex or the virus infection alone is sufficient to cause IgAN. The mechanism of IgAN pathogenesis is still not well known.

In addition, a series of studies have shown that 40–60% of IgAN cases recur even after healthy kidney transplantation, whereas the deposited IgA gradually disappears in glomeruli when the IgAN kidney is transplanted to healthy individuals [18, 19, 20]. These evidences from kidney transplantation strongly support that IgAN is an autoimmune disease rather than a kidney‐oriented disease. Systematic dissection of immune composition may contribute to a better understanding of the alternation of the immune system in IgAN.

In this study, we applied immune repertoire sequencing technology to systematically analyze the complementary determining region 3 (CDR3) sequences of all the B cell receptors (BCRs) and evaluated the antibody CDR3 length distribution, diversity, percentage of each subtype and overlapping of CDR3 sequences among different isotypes, particularly for IgA, IgG and IgM in both patients with IgAN and the normal control (NC) group. By comparing data from IgAN and NC groups, we try to answer the following questions: (a) What is the average CDR3 length, and does it change the immune diversity in IgAN? (b) What is the composition of different antibody subtypes in IgAN? (c) How about the shared sequences among different subtypes? Is this related to class switching distortion in IgAN? (d) Is there any difference in CDR3 length distribution and amino acids usage preferences in IgAN? The study aimed to provide important clues for a therapeutic rationale in a new way.

## Materials and methods

### Patients

Whole blood sample and renal biopsy of 13 patients with IgA, ranging in age from 22 to 62 (median 26) years, were collected during August 2014 and October 2014, and seven healthy candidates were recruited into this study (Table S1). The study was performed according to the principle of the declaration of Department of Nephrology, The 6th Affiliated Hospital of Shenzhen University Health Science Center, China. The study protocol was approved by the medical ethics committee of this university. All participants gave written informed consent. The blood, urine analysis and renal biopsy were tested from the main components according to the methods recommended by the World Health Organization [21]. The study was performed according to the guidelines of the Department of Nephrology, The 6th Affiliated Hospital of Shenzhen University Health Science Center. All patients gave written, informed consent. The IgAN and NC groups fulfilled the classification criteria, as listed in Table S1.

### Peripheral blood mononuclear cell isolation

Peripheral blood mononuclear cells (PBMCs) were isolated using LymphoPrep™ (Axis‐shield, Dundee, Scotland, UK) with slight modification of the manufacturer's instructions. In brief, 10 mL of blood was diluted with an equal volume of PBS, loaded carefully onto 5 mL of LymphoPrep (volume of sample to LymphoPrep media=2 : 1) and centrifuged at 600 ***g*** for 20 min by swing angle centrifuge. The thin PBMC layer at the sample–medium interface was carefully aspirated, transferred to a new tube and washed twice with PBS at 300 ***g*** for 10 min. The purified PBMCs were pelleted for RNA extraction.

### RNA extraction

The total RNA was extracted using the Qiagen RNeasy Mini Kit (Qiagen, Hilden, Germany) according to the manufacturer's instructions. Around 5 × 10^6^ PBMCs were lysed thoroughly by 600 mL of RLT buffer (with β‐mercaptoethanol), and one volume of 70% EtOH was added to the homogenized lysate and mixed well by pipetting. Seven hundred milliliters of the sample was loaded to the RNeasy spin column in a 2‐mL collection tube and centrifuged for 15 s at 8000 ***g***; then we added the remaining lysate to the same column and repeated the centrifuge. Then the column was washed by 700 mL of RW1 buffer once and 500 mL of RPE buffer twice at 8000 ***g***. Finally, the RNA was collected in a new 1.5‐mL collection tube by adding 30 mL of RNase‐free water directly to the spin column and centrifuging at 8000 ***g***. RNA concentration was determined by Qubit 2.0, and the samples were stored at −80 °C in a refrigerator or processed for reverse transcription (RT) immediately.

### Primer design

Human Ig heavy‐chain gene sequences were downloaded from the international ImMunoGeneTics information system (IMGT; http://www.imgt.org/) [22]. A relatively conserved region in‐framework region 3 (FR3) upstream of Ig heavy‐chain variable region gene (IGHV) CDR3 was selected for the putative forward primer region. Twelve forward primers corresponding to the majority of the V gene sequence family were designed. Similarly, five reverse primers corresponding to five isotypes (*IgG, IgA, IgM, IgD* and *IgE*) from the antibody constant region were modified from previous publications [23]. The forward and reverse primers were analyzed by Oligo 7.0 (Molecular Biology Insights, Colorado Springs, CO, USA) and MFEprimer‐2.0 (MFEprimer, Shanghai, China) software for primer dimer and loop structures. Minor changes in the sequences were made for low‐quality primers. The primer sequences are shown in Table S4.

### Primer and PCR amplification validation

The sequencing data were analyzed using a similar method as proposed by Robins *et al*. [24] to validate the PCR bias [23]. To prepare the BCR library, first, we prepared the BCR forward primer pool and BCR reverse primer pool by mixing equal volume of 12 forward primers and 5 reverse primers, respectively. The final concentration of forward primer pool (10/12 mm for each forward primer) and reverse primer pool (10/5 mm for each reverse primer) was set to 10 mm. cDNA was synthesized with RevertAid H Minus First Strand cDNA Synthesis Kit (Thermo Fisher, Waltham, MA, USA). RT was performed by mixing 10 mL of RNA (about one‐third of the RNA purified from each sample) and 2 mL of 10 mm reverse primer pools, denaturing at 65 °C for 5 min and chilling immediately on ice. Then 4 mL of 5× reaction bmuffer, 2 mL of 10 mm dNTP, 1 mL of RNase inhibitor and 1 mL of reverse transcriptase were added to the mixture to make a final volume of 20 mL and processed for the RT. The reactions were incubated at 42 °C for 1 h and then deactivated at 70 °C for 5 min and held at 4 °C. For each sample, the RT product is later used in multiplex PCR (Qiagen). In a 50‐mL reaction system, we added 25 mL of 2× Qiagen Multiplex PCR Master Mix, 5 mhL of 5× Q‐solution, 2 mL of BCR forward primer pool and 18 mL of RT product in a 0.2‐mL PCR tube and began with an initial denaturation at 95 °C for 15 min, followed by denaturation at 94 °C for 15 s, annealing of primer to DNA at 60 °C for 3 min and extension at 72 °C for 5 min and held at 4 °C. PCR products were cleaned with Agencourt Ampure XP beads (Beckman Coulter, Krefeld, Germany) following the manufacturer's instructions. The beads were balanced at room temperature for at least 30 min and mixed thoroughly. In each PCR tube, we added 1.8 volume of PCR product and incubated at room temperature for 5 min.

The DNA fragments larger than 100 bp are collected by paramagnetic beads and separated by Dynal Invitrogen bead separation (Invitrogen, Carlsbad, CA, USA). The beads are then washed by 80% EtOH to remove contaminants. Finally, the DNA fragments were eluted by distilled water and transferred to a newgt tube. Sequencing indices and adaptors were added to the BCR libraries in the second round of PCR. In a 50‐mL system, we added 25 mL of 2xphusion mix, 1 mL of P1 adaptor, 1 mL of Index and 23 mL of purified DNA from the last step. The PCR conditions for adding indices were 98 °C for 1 min, followed by 25 cycles of 98 °C for 20 s, 65 °C for 30 s and 72 °C for 30 s, with a final extension for 7 min at 72 °C. The library was separated on an agarose gel, and the target region was isolated and cleaned by QIAquick Gel Extraction Kits (Qiagen). The library was quantitated by Agilent 2100 bioanalyzer (Agilent, Palo Alto, CA, USA) and Qubit 2.0 (Thermo Fisher), then sequenced by Illumina sequencer (Illumina, San Diego, CA, USA).

### Data analysis

A total of 18 976 912 pair‐end reads were generated by the Illumina sequencer. We used FLASH software to merge overlapping paired‐end reads, and obtained 16 376 727 raw reads [25]. The merged sequence reads were aligned to V, D and J gene germline references using IgBLAST, and the constant region was mapped to IgA, IgD, IgE, IgG and IgM, respectively [26, 27]. The reference sequences of V, D and J gene germlines were obtained from IMGT. Reads with low alignment identity (<70%) to germline references were excluded. After read filtering, 11 114 426 reads were obtained for further analysis. The starting and ending position of the CDR3 region, reading frame and productivity were identified according to the definition of IMGT [22]. The hypermutation level for IgAN samples is analyzed by the newly developed software mixcr (mixcr: a universal tool for fast and accurate analysis of T cell receptor and BCR repertoire sequencing data). Normalized Gini, Shannon entropy, Simpson and G50 indices were used to evaluate the diversity of the antibody repertoire. Unpaired two‐tailed *t*‐test was applied to calculate the *P* value between IgAN and NC. Meanwhile, the *P* values were calculated using Wilcoxon rank sum and signed rank tests (wilcox.test).

## Results

### CDR3 length distribution and amino acids usage bias in IgAN

The CDR3 is the place where the recombination, random nucleotide addition or deletion of V, D and J region elements occurs, which contributes a lot to the diversity of antigen specificities. The length and amino acids composition are important determinants of BCR repertoire diversity.

**Fig. 1:**
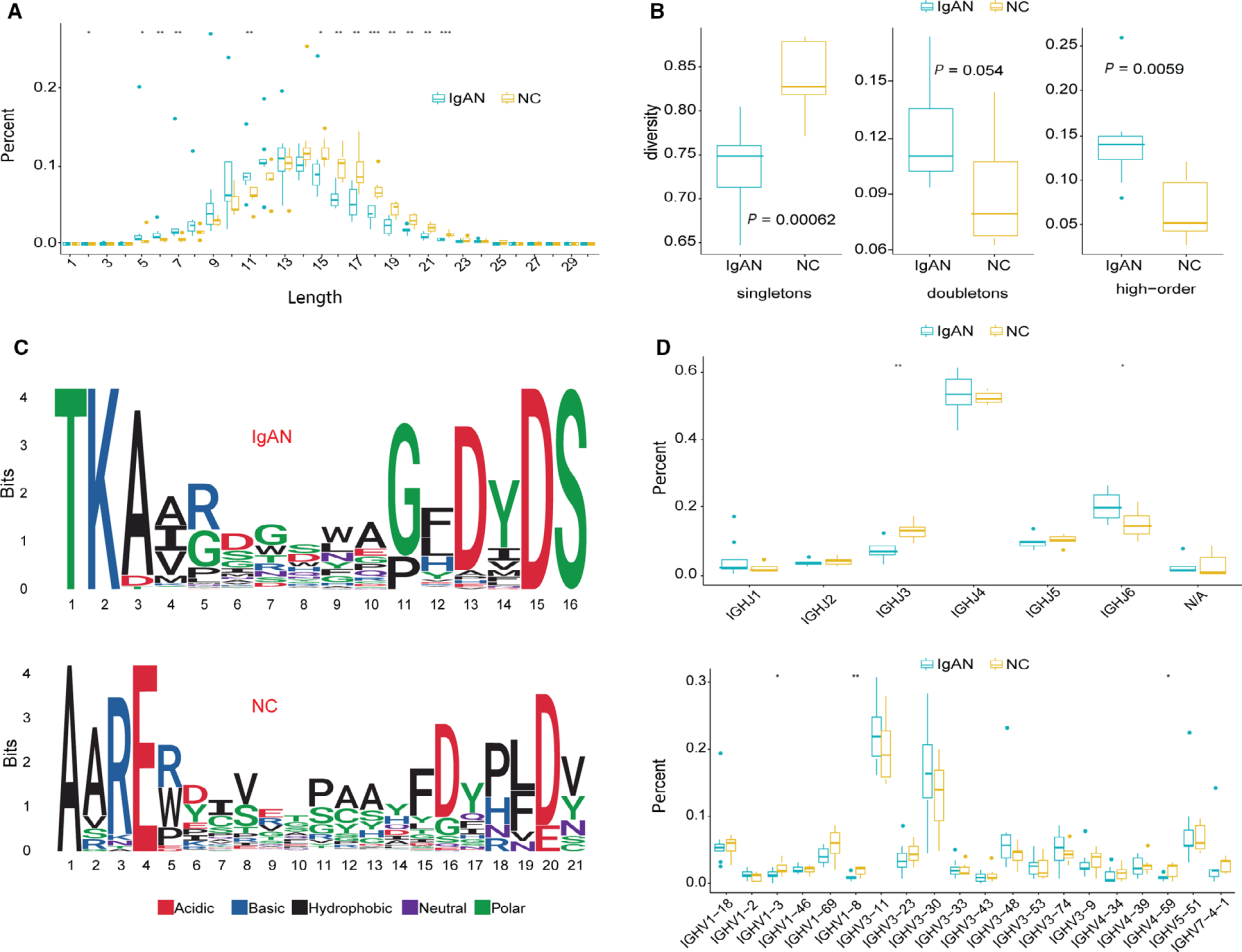
CDR3 analysis of IgAN. (A) The length distribution in patients with IgAN and NCs. *P < 0.05, **P < 0.01, and ***P < 0.001 indicate significant differences of individual length between IgAN and NC (wilcox.test). (B) Unique clone abundance of the CDR3 sequences. (C) Weblogo of amino acids sequence of IgAN and NC. (D) V, J gene usage preference of IgA in IgAN and NC.

First, we analyzed the CDR3 length distribution in IgAN and NC groups (Table S2). The results showed the IgAN group has shorter CDR3 amino acids length compared with that in the NC group (Figs 1A and S1; Tables S1 and S2). The dominant amino acids sequences are clustered within 5–12 amino acids, in particular, the lengths at 5–7 and 11 are significantly increased in IgAN, and the length between 15 and 22 is significantly down‐regulated in IgAN (Fig. 1A). The pattern of the amino acids usage is different in two groups. The top 10 CDR3 amino acid sequences of IgAN and NC are shown in Table S3 and Fig. S1A. The shortened average length of CDR3 sequence in IgAN also affected the diversities, which results in the decreased potency of the immune response in IgAN.

Next, we examined the repertoire clonality frequency by grouping them into three groups as singletons, doubletons or high‐order based on their abundance of the unique CDR3 amino acids sequences (Fig. 1B): antibody sequences with only one unique sequence are decreased (as shown as singletons), whereas the doubletons and high‐order (3+) CDR3 sequences are significantly increased in IgAN, indicating the diversity of BCRs is decreased in IgAN. The IgAN group contains two dominant clones with a very short amino acids sequence, but there are no dominant clones observed in the NC group (Table S3).

Because both the CDR3 amino acids sequence length and the top 10 abundant CDR3 sequences are quite different in the two groups, as shown in Fig. 1A,B, we plotted the weblogo chart in the patients with IgAN and NCs to clearly present the difference of the amino acids usage preference. IgAN samples have a similar amino acids sequence, for example, the IgAN samples start with ‘TK’ and end with ‘DS’, whereas NC samples are more diverse (Fig. 1C). However, to make a solid conclusion, more IgAN samples are required.

Next, we performed a statistical analysis of different V and J gene usages in the two groups. IGHJ3 (JH3) are significantly decreased and IGHJ6 (or JH6) are significantly increased in the IgAN group. For the V genes, IGHV1–3, IGHV1–8 and IGHV4–59 are significantly decreased in IgAN (Figs 1D and Fig. S2–S4).

Overall, these results illustrate that the length of CDR3 in IgAN is shortened globally compared with that in control. A decrease in CDR3 results in a decrease in the potential of BCR diversity.

### The clonal diversity is significantly decreased in IgAN

**Fig. 2:**
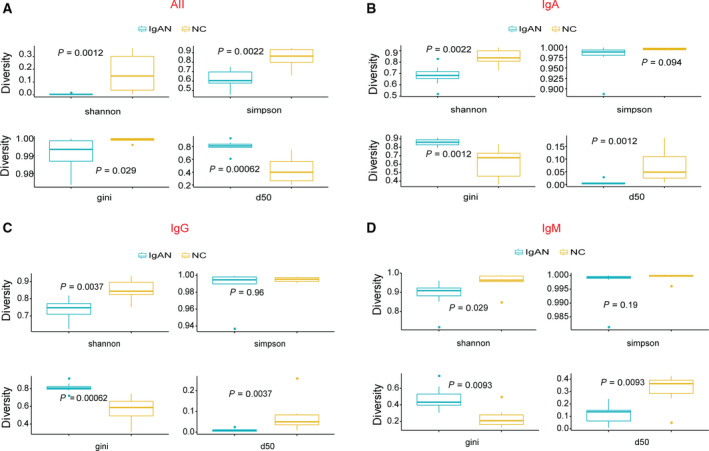
CDR3 diversity analysis. Shannon, Simpson, Gini and Diversity 50 indices in all antibodies (A), IgA (B), IgG (C) and IgM (D). Significant differences between patients with IgAN and NCs are identified. We identified the middle 50% of data, as well as the extreme points in the boxplot. The median is shown as the bold line in the box, and we set the lower quartile at 25th percentile and upper quartile at 75th percentile.

To verify whether the decrease of CDR3 length influences the diversity of IgAN, we used a series of statistical models, including Diversity 50 (D50) index, Shannon entropy, Simpson entropy and Gini test, to systematically evaluate the diversity of CDR3 sequence in IgAN and NC. The diversity of immune repertoire usually can partially reflect the efficiency of the adaptive immune response. We calculated the indices for all of the subtypes (sample ID ‘all’, Fig. 2A) and three other major isotypes, including IgA (Fig. 2B), IgG (Fig. 2C) and IgM (Fig. 2D). D50 index is the calculated percentage of dominant unique clones, accumulative reads of which make up for 50% of the total CDR3 sequences (ranges from 0 to 50 in theory) [28]. It is directly and positively related to diversity. Our data suggest that the diversity in IgAN is significantly decreased. Similar results are also obtained through normalized Shannon entropy and Gini *t*‐test (Fig. 2). However, the result of Simpson *t*‐test indicates that the difference between the two groups is not significant. The Shannon index puts more weight on richness (how many different species are there, including the rare one). Compared with Shannon entropy that focuses on the uniform of individual sequences in one sample, the Simpson *t*‐test is usually determined by the dominant clones (rare species do not make much difference). The result may imply that the number of dominant clones is similar. However, the diversity is much higher in NCs.

To clarify how the diversity is affected in the different subtypes of antibodies, we performed statistical analysis for the three most abundant antibodies, including IgA, IgG and IgM (IgE and IgD are rare in PBMCs). The diversity is significantly lower in patients with IgAN than in the NC group, particularly for IgA and IgG (Fig. 2B,C). Because the naive B cells express only IgM, the reduced diversity of IgM can reflect the lower diversity of naive B cells in patients with IgAN, which may indicate less potent of an immune response for patients with IgAN.

These results may suggest that expression patterns of IgM, IgG and IgA were all distorted in IgAN. Decreases in the diversity of all the subtypes may be because of the restricted clone expansion of both IgG and IgA, which can specifically target to the altered structure in IgAN, such as underglycosylated IgA1. Because the IgM is also affected, it indicates that the alteration of clonal diversity in IgAN may start before the antibodies encounter their antigens and will be enlarged further after that.

### The percentages of IgA and IgG subtypes are elevated in IgAN

From the earlier diversity analysis, we observed that the overall diversity of IgA, IgG and IgM was significantly decreased in IgAN, which may be caused by several reasons: decrease of the total number of white blood cells, for example, by chemotherapy, or overexpression of one or a few B cell clones.

**Fig. 3:**
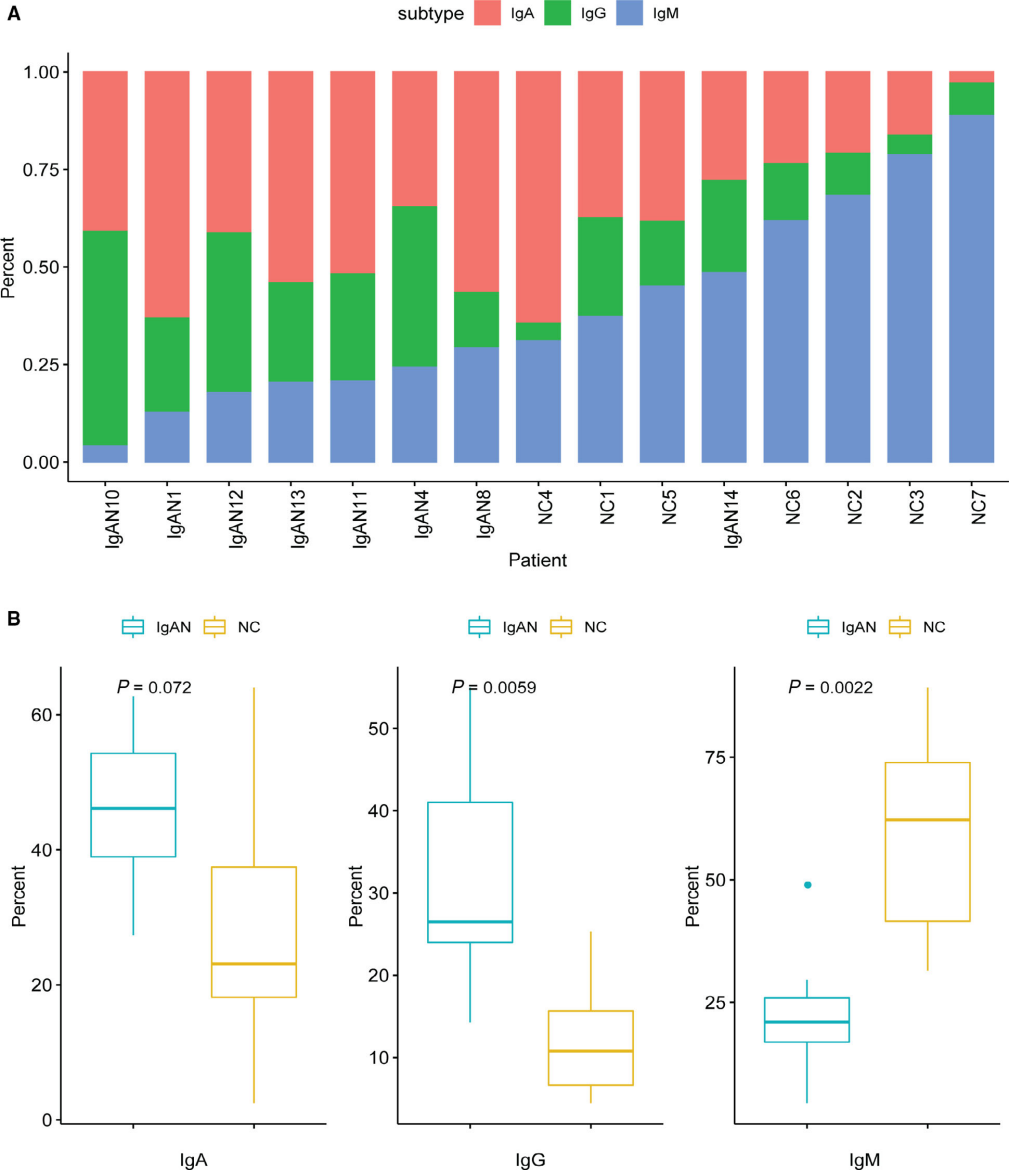
Isotype distribution in patients with IgAN and NCs. (A) Bar chart shows the composition of different antibody isotypes in 13 patients and 7 NCs. Although the IgM is dominated in NCs, IgA and IgG are increased in patients with IgAN. (B) Boxplot showing the percentage of three dominant antibody isotypes, IgA, IgG and IgM, in patients with IgAN and NCs. The *P* value indicated that there is a significant difference in the amount of isotypes in patients with IgAN and NCs (wilcox.test).

To reveal the detailed mechanisms in IgAN, we examined the percentage of each antibody subtype in IgAN and NC samples (Fig. 3A). The relative expression of IgA and IgG genes is significantly induced in IgAN compared with NC [IgAN: IgA median, 0.45 (0.24–0.69), IgG median, 0.36 (0.16–0.55); control: IgA median, 0.23 (0.05–0.42), IgG median, 0.14 (0.06–0.220)], whereas the expression level of IgM is decreased in patients [IgAN: IgM median, 0.17 (0.06–0.33); control: IgM median, 0.80 (0.40–0.80)], suggesting that more IgM is switched to IgA or IgG probably by the dysfunction of certain crucial factors in the class‐switching pathways in IgAN. Statistical analysis was also performed to confirm the change between these two groups (Fig. 3B). An almost 2.5‐fold increase of IgA and IgG and 3‐fold decrease of IgM in patients with IgAN were observed.

Because of the important role of each subtype in maintaining primary immune status, as well as immune response, our data imply that the immune status and immune responses may change a lot in patients with IgAN.

**Fig. 4:**
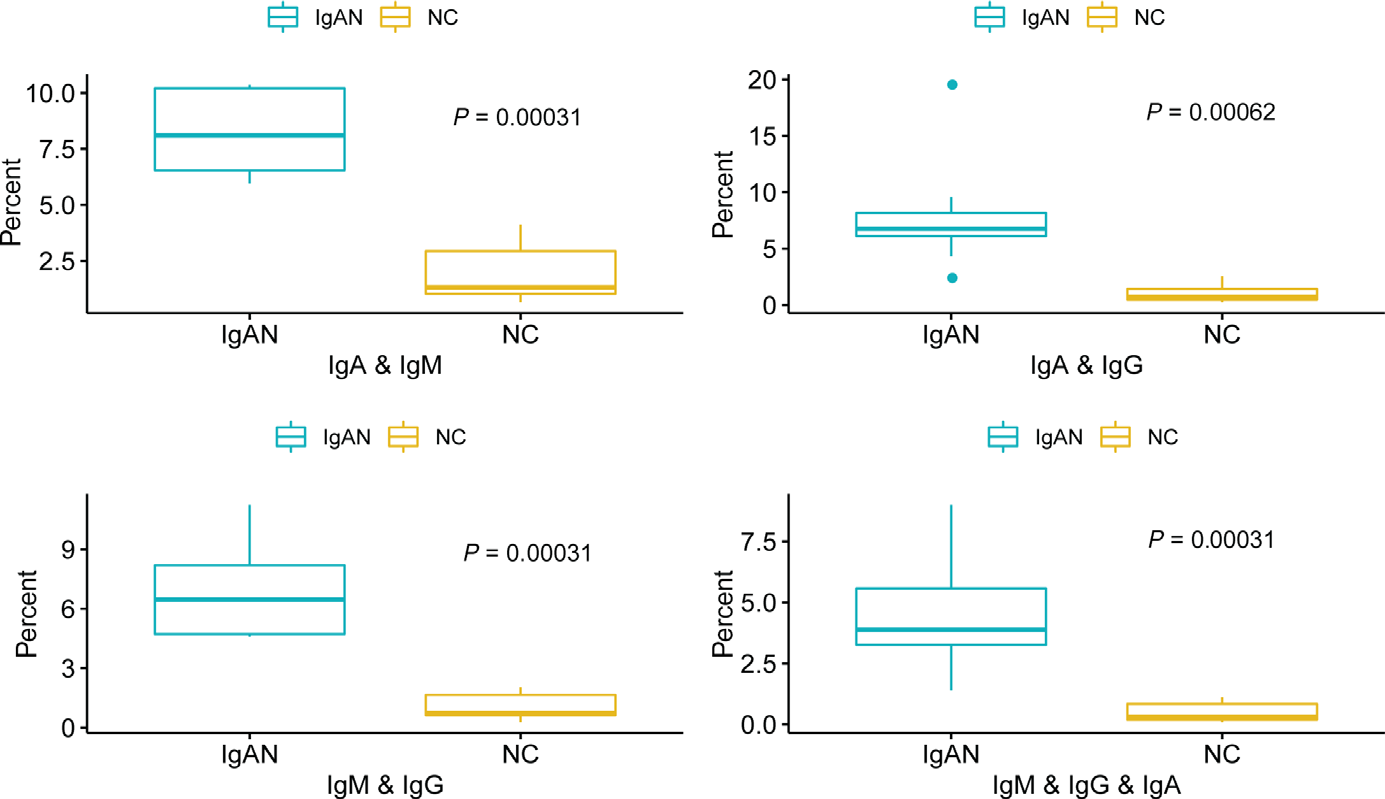
Sharing of CDR3 sequences. The overall sharing rate between each of IgM, IgA and IgG in patients with IgAN and NCs (*P* < 0.001, wilcox.test).

There are overlapping CDR3 sequences among different antibody subtypes because IgA and IgG are switched from IgM after they encounter the antigens. We further compared the shared CDR3 gene sequences of different antibody subtypes in the IgAN group and also the NC group by three combinations, including IgA and IgM, IgA and IgG and among IgA and IgG and IgM all three subtypes (Fig. 4). All the naive B cells express only IgM, and when the B cells encountered the antigens, some IgMs can transform to IgA and IgG. We observed a dramatic increase of the percentage of shared CDR3 sequences between IgM and IgA in IgAN compared with that in the control group (6.8% in IgAN versus 1.5% in control). There is a more than 4‐fold increase in the shared sequences in IgAN. The common CDR3 in IgM/IgG/IgA is also increased more than 7‐fold (3.6% in IgAN versus 0.5% in control) (Fig. 4). The overlapping of the CDR3 sequences in NCs is much lower. In IgAN, the immune system is activated; therefore, a large percentage of sequences is shared in IgM, IgG and IgA.

## Discussion

Different isotypes of antibodies are adapted to function in different compartments of the body, where they will interact with different effector cells and molecules. In theory, the majority of the IgA is secreted into the mucosal and defends against the pathogens. Disruption in the antibody functions can be observed by the expression level and pattern in peripheral blood. However, there is still no treatment known to modify mesangial deposition of IgA due to lack of a complete understanding of the pathogenesis of IgAN. It is important to further investigate the mechanism of the deposited IgA. Several observations contribute to the idea that the so far unresolved pathogenesis of IgAN involves the immune system rather than specific renal factors.

We here applied immune repertoire sequencing technology to parallel the analyzed CDR3 region of all the BCRs, including all five antibody subtypes: *IgA, IgG, IgM, IgE* and *IgD*, in 13 patients with IgAN and 7 NCs without IgAN. We observed a significant decrease of the CDR3 length in IgAN compared with that in the control group (Fig. S1). Nevertheless, there are more highly expanded clones in IgAN, almost twice that in the control. The amino acids usage also showed different patterns in IgAN. We also observed a high rate of some dominant CDR3 overlapped in IgA, IgG and IgM; it is possible that for a given V region, it can become associated with any C region through isotype switching. To identify any disease‐specific V or J gene usage in IgAN, we then investigated the germline gene usage in IgAN and NC samples (Fig. S2 and Table S5). The overall pattern of VJ gene usage is very similar (Fig. S2C); however, the JH6 genes are significantly increased in IgAN (Fig. 1D). When we compared the CDR3 length with and without JH6, we found that sequences with JH6 are on average longer than sequences not composed of JH6 (Fig. S3), but the overall length of CDR3 in IgAN is much shorter, which makes it a little complex to understand the role of overuse of JH6 in IgAN. The D gene usage of IgAN and NC are also studied (Fig. S4). The progeny of a single B cell can produce antibodies, all specific for the same eliciting antigen, which provide all of the protective functions appropriate for each body compartment.

Several statistical indices, including Shannon entropy, D50 and Gini test, all showed significant differences in the diversity of IgG, IgM and IgA antibodies to controls (Figs 2 and Fig. S7). The percentages of IgA and IgG are increased and IgM is decreased in IgAN.

Nevertheless, despite the dramatic increase in IgA proportion in total BCRs in patients with IgAN, very few mutations and diversity were observed in the CDR3 of IgA (or IgG) as compared with the high abundance of germline sequences or the high rate of somatic hypermutation for B cells when activated by foreign antigens. The hypermutation level of the top 20 clones in IgAN was evaluated (Fig. S5). The mutation rate of V, D, J gene and total rate are calculated, with an average of overall mutation rate around 1.11 mutations per sequence in our study, in particular, the mutation rate for J gene is higher than V or D genes (Fig. S6). The BCR locus undergoes an extremely high rate of somatic mutation that is at least 10^5^‐ to 10^6^‐fold greater than the normal rate of mutation across the genome during proliferation. It may result from the low affinity of the IgAs to antigens in IgAN as reported by Layward *et al*. [29]. Combining these evidences, it is possible that these IgAs are not fully maturated and with low affinity, which can explain the predominant deposition of IgA in this disease. These findings suggest that the BCR immune repertoire is altered dramatically in IgAN, characterized by the shortened CDR3 length, as well as decreased overall diversity of CDR3.

## Conflict of interest

The authors declare no conflict of interest.

## Author contributions

ZL designed the study. YT and XL recruited the normal candidates, while SZ recruited the patients and recorded the participants’ information. All authors participated in the experiments in this study. ZL, JZ, YT and XZ further performed data analysis. ZL, LZ and XZ wrote the manuscript. Finally, ZL, LZ, XZ and SZ revised manuscript.

## Supporting information


**Table S1.** Clinical and biological data. A total of 13 patients with IgAN were recruited into the study (the information of seven normal candidates was not shown). Finally, eight IgAN samples and seven NC samples were qualified and later included in our statistical analysis.
**Table S2.** Sequence statistics (reads per unique CDR3). Reads abundance in eight IgAN samples and seven NC samples.
**Table S3.** Top 10 shared CDR3 amino acids sequence in IgAN and control.
**Table S4.** The sequence information of PCR primers.
**Table S5.** J‐gene usage.
**Fig. S1.** Skewed CDR3 length distribution in patients with IgAN and controls. (A) CDR3 distribution and top 10 amino acids sequences in IgAN and NC. (B) Significance analysis of the length distribution between IgAN and control (*P < 0.05).
**Fig. S2.** V and J gene usage in IgAN. (A) V and J gene usage of the variable region in IgAN and NC. (B) V gene usage preference in all antibody repertoire and IgA, IgM and IgG, respectively. (C) J gene usage preference in all antibody repertoire and IgA, IgM and IgG, respectively.
**Fig. S3.** The average length of CDR3 with or without JH6. The average length of CDR3 is around 17.7, and the average length of CDR3 without JH6 is around 14.4. There are significant differences between JH6^+^ and JH6^−^ with *P* < 0.001 (*P* = 2.2e−16).
**Fig. S4.** D gene usage in IgAN.
**Fig. S5.** Number of hypermutation level of top 20 clones in IgAN.
**Fig. S6.** V, D and J gene mutation rate in IgAN.
**Fig. S7.** Correlation of CDR3 diversity versus other clinical markers. Four statistical indices, including D50, Shannon, Simperson and Gini, show that several clinical markers are positively correlated with the CDR3 diversity (blue spots), whereas other markers are negatively correlated with the CDR3 diversity (red spots).

## Data Availability

Data are accessible through NCBI’s GEO Series accession number PRJNA668873 (https://dataview.ncbi.nlm.nih.gov/object/), and the data are also available from the corresponding author upon reasonable request.
