# Supplementary material for: CDR3 sequences in IgA nephropathy are shorter and exhibit reduced diversity
Source: FEBS Open Bio. 2020 Nov 20;10(12):2702–11. doi: 10.1002/2211-5463.13006 (PMC7714077; doi:10.1002/2211-5463.13006)

Supplementary materials

Supplementary Table legends:

Table S1. Clinical and Biological Data. Total 13 IgAN patients are recruited in the study (the information of 7 normal candidates was not showed). Finally, 8 IgAN samples and 7 NC samples are qualified and later included in our statistic analysis.

Table S2. Sequence Statistics (reads/unique CDR3). Reads abundance in 8 IgAN samples and 7 NC samples.

Table S3. Top 10 Shared CDR3 Amino-Acids Sequence in IgAN and Control.

Table S4. The Sequence Information of PCR Primers.

Table S5. J-gene Usage.

Supplementary Figure legends:

Figure S1. Skewed CDR3 length distribution in IgAN and controls. (A) CDR3 distribution and Top 10 amino acids sequences in IgAN and NC. (B) Significance analysis of the length distribution between IgAN and control (*P<0.05).

Figure S2. V and J gene usage in IgAN. (A) V and J gene usage of the variable region in IgAN and NC. (B)V gene usage preference in all antibody repertoire, and IgA, IgM, IgG, respectively. (C) J gene usage preference in all antibody repertoire, and IgA, IgM, IgG, respectively.

Figure S3. The average length of CDR3 with or without JH6.

The average length of CDR3 with is around 17.7, and the average length of CDR3 without JH6 is around 14.4. There are significant differences between the JH6+ and JH6- with p-value < 0.001(p-value=2.2e-16).

Figure S4. D gene usage in IgAN

Figure S5. No. of Hypermuation Level of Top20 clones in IgAN

Figure S6. V, D and J gene mutation rate in IgAN

Figure S7. Correlation of CDR3 diversity vs other clinical markers. Four statistical indices including d50, Shannon, Simperson and Gini show that several clinical markers are positively correlated with the CDR3 diversity(blue spots), while other markers are negatively correlated with the CDR3 diversity(red spots).

Supplementary Tables:

Table S1. Clinical and Biological Data

ID	Gender	Age	Course of disease	Symptoms	Diagnosis
**IgA-1**	M	24	1 month	urine occult blood, albuminuria	Lee grade, III
**IgA-2**	M	25	1 month	abnormal in Urine Analysis，Fever 3days	Lee grade, III
**IgA-3**	F	27	2 months	hematuria, albuminuria	Lee grade, III
**IgA-4**	F	62	2 years	hematuria, albuminuria	Lee grade, III
**IgA-8**	F	23	2 months	abnormal in Urine Analysis	Lee grade, II
**IgA-10**	M	22	3 weeks	hematuria，fever 3days and gross hematuria	Lee grade, II
**IgA-11**	M	38	3 months	abnormal in Urine Analysis	Lee grade, IV
**IgA-12**	F	46	8 years	albuminuria	Lee grade, IV
**IgA-13**	M	35	1 month	abnormal in Urine Analysis	Lee grade, III
**IgA-14**	M	26	4 months	abnormal in Urine Analysis	Lee grade, II
**IgA-15**	M	25	2 years	gross hematuria	Lee grade, III
**IgA-16**	M	33	1 month	abnormal in Urine Analysis	Lee grade, III
**IgA-17**	M	31	1 year	abnormal in Urine Analysis	Lee grade, II
**NC1**	F	23	Healthy control	No medication	
**NC2**	F	25	Healthy control	No medication	
**NC3**	F	27	Healthy control	No medication	
**NC4**	F	25	Healthy control	No medication	
**NC5**	M	26	Healthy control	No medication	
**NC6**	F	23	Healthy control	No medication	
**NC7**	M	27	Healthy control	No medication	

Table S2. Data Statistics

Sample ID	All reads	Not qualified	Not cdr3 aa	Group
IgAN1	432619	0.259119456	7.139307335	IgAN
IgAN10	555245	0.237372691	15.50540752	IgAN
IgAN11	485496	0.567254931	5.311063325	IgAN
IgAN12	503726	0.503051262	5.896658104	IgAN
IgAN13	1472111	0.038991625	8.408469198	IgAN
IgAN14	1398189	0.074095848	8.858387528	IgAN
IgAN4	833151	0.911719484	5.625870941	IgAN
IgAN8	525198	0.67498353	15.78871207	IgAN
NC1	115656	6.080099606	25.17379124	NC
NC2	127082	3.832958247	12.8971845	NC
NC3	53357	0.001874168	4.818486796	NC
NC4	192382	0.266656964	17.74282417	NC
NC5	396471	0.015385741	4.422265437	NC
NC6	65930	0.00303352	5.235856211	NC
NC7	202129	0.00692627	4.441717913	NC

Table S3. Top10 CDR3 aa in IgAN and Control

Ranking	CDR3 aa in IgAN	CDR3 aa in Control
1	TIMES	ARWDCSRTSCHQFDQ
2	ARPIGGS	ARDRGRWYQLLYDPLDV
3	GGTSNGWHEIDS	ARYVVLSPALGGSRLDY
4	ASGQQLGH	PKIGFGYSYGGGLDV
5	ATGQQLGY	AKDRSPRYDVMTGGLDS
6	APGRQLGDY	ARYIVESPAAYFDY
7	AMGMNSGPFDY	ARRKCSSTSCYDDY
8	GGTMNGWHEIEA	ARRISSTGRSSAFDI
9	AIGSGHFQH	ARSYCSSTSCHATGYFDY
10	VRDDSWAFDY	ARYIVEQPAAHFDH

Table S4. Primer Sequences for BCR amplification

Primer Name		Primer Sequences
Forward Primers	IGHV1-18	CAGACGTGTGCTCTTCCGATCTAGNNNNNNNNAGAGTCACCATGACCACAGAC
Forward Primers	IGHV1-2/ 1-46	CAGACGTGTGCTCTTCCGATCTAGNNNNNNNNAGAGTCACCAKKACCAGGGAC
Forward Primers	IGHV1-24 -8N	CAGACGTGTGCTCTTCCGATCTAGNNNNNNNNAGAGTCACCATGACCGAGGAC
Forward Primers	IGHV1-3/ 1-45	CAGACGTGTGCTCTTCCGATCTAGNNNNNNNNAGAGTCACCATTACYAGGGAC
Forward Primers	IGHV1-69 /1	CAGACGTGTGCTCTTCCGATCTAGNNNNNNNNAGAGTCACGATWACCRCGGAC
Forward Primers	IGHV1-8	CAGACGTGTGCTCTTCCGATCTAGNNNNNNNNAGAGTCACCATGACCAGGAAC
Forward Primers	IGH2-70/ 26/5	CAGACGTGTGCTCTTCCGATCTAGNNNNNNNNACCAGGCTCACCATYWCCAAGG
Forward Primers	IGHV3	CAGACGTGTGCTCTTCCGATCTAGNNNNNNNNGGCCGATTCACCATCTCMAG
Forward Primers	IGH4	CAGACGTGTGCTCTTCCGATCTAGNNNNNNNNCGAGTCACCATRTCMGTAGAC
Forward Primers	IGHV5-51	CAGACGTGTGCTCTTCCGATCTAGNNNNNNNNCAGCCGACAAGTCCATCAGC
Forward Primers	IGHV6-1	CAGACGTGTGCTCTTCCGATCTAGNNNNNNNNAGTCGAATAACCATCAACCCAG
Forward Primers	IGHV7	CAGACGTGTGCTCTTCCGATCTAGNNNNNNNNGACGGTTTGTCTTCTCCTTG
Reverse Primers	IgM-8N-R	CTACACGACGCTCTTCCGATCTNNNNNNNNGGGAATTCTCACAGGAGACG
Reverse Primers	IgE-8N-R	CTACACGACGCTCTTCCGATCTNNNNNNNNGAAGACGGATGGGCTCTGT
Reverse Primers	IgD-8N-R	CTACACGACGCTCTTCCGATCTNNNNNNNNGGGTGTCTGCACCCTGATA
Reverse Primers	IgA-C36-8 N-R	CTACACGACGCTCTTCCGATCTNNNNNNNNGCTCAGCGGGAAGACCT
Reverse Primers	IgG-C22- 8N-R	CTACACGACGCTCTTCCGATCTNNNNNNNNAGGGYGCCAGGGGGAAG

Table S5. Differential expressing J genes in IgAN

Ig type	Increased in IgAN	Decreased in IgAN
**ALL**	IGHJ6	IGHJ3
**IgG**	IGHJ6	IGHJ3
**IgA**	NULL	IGHJ3
**IgM**	IGHJ6	IGHJ3

Supplementary Figures:

Fig. S1

(A)

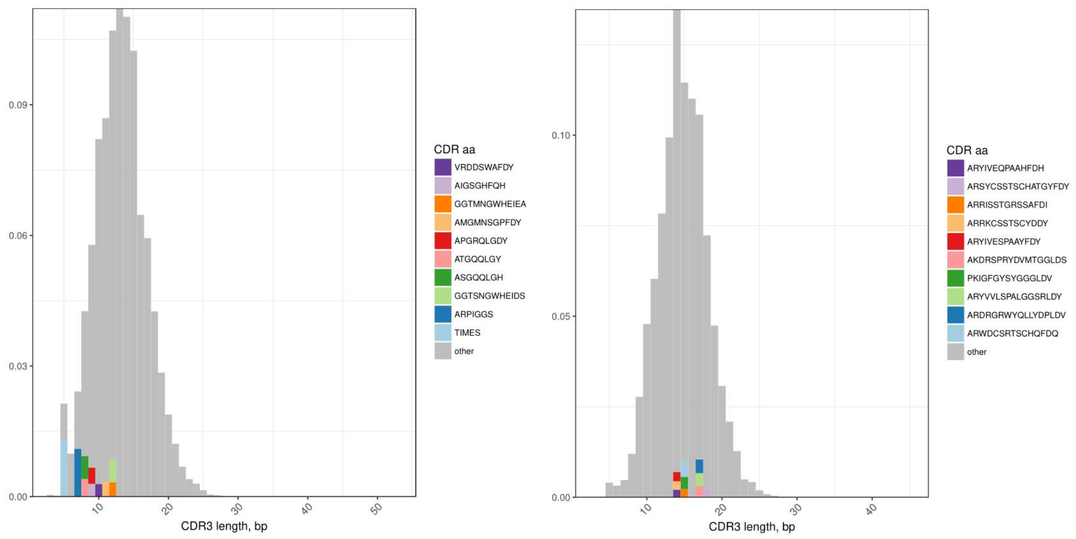

Top 10 amino acids in IgAN　　　　Top 10 amino acids in NC

(B)

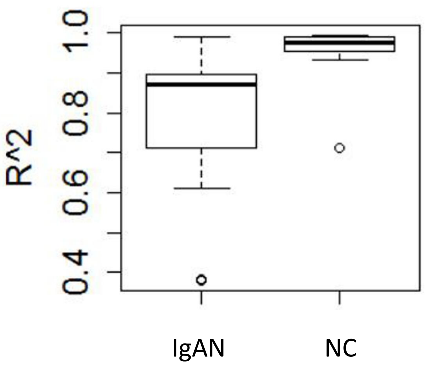

Fig. S2

(A)

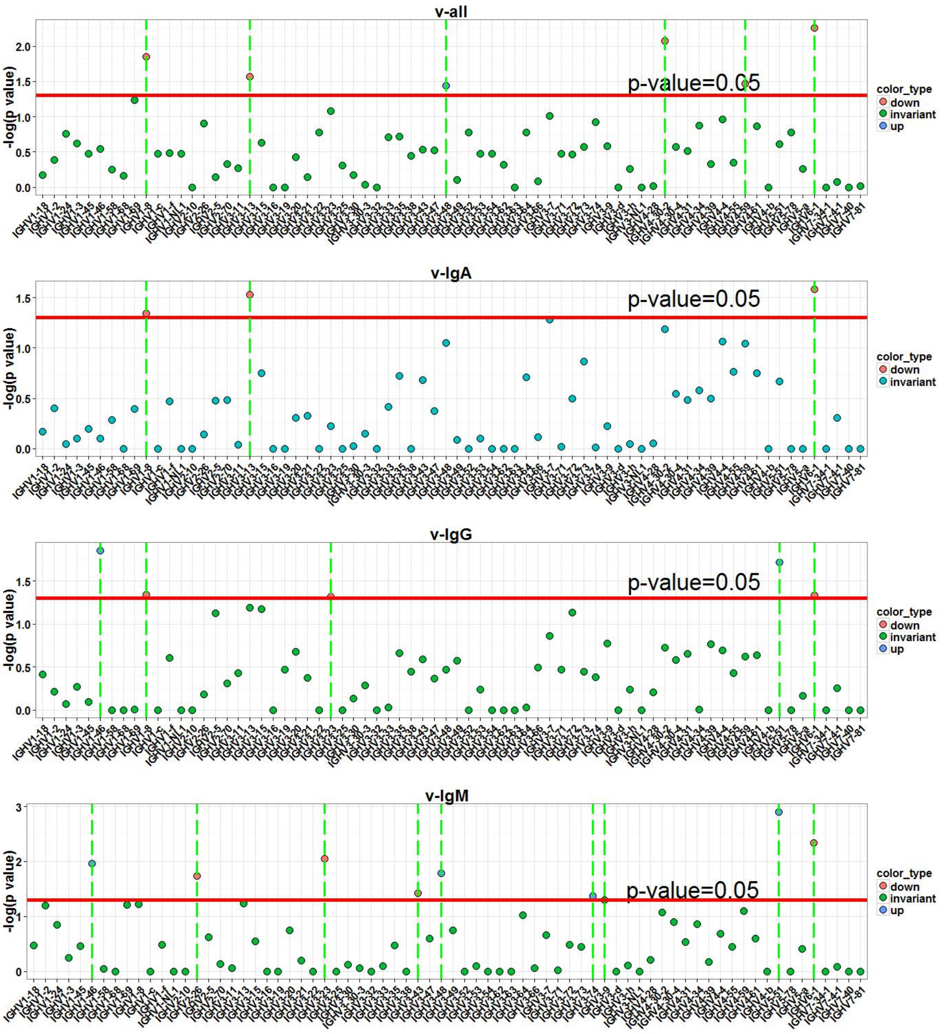
v-all
v-IgA
v-IgG
v-IgM
-log(p value)
p-value=0.05
color_type
down
invariant
up

(B)

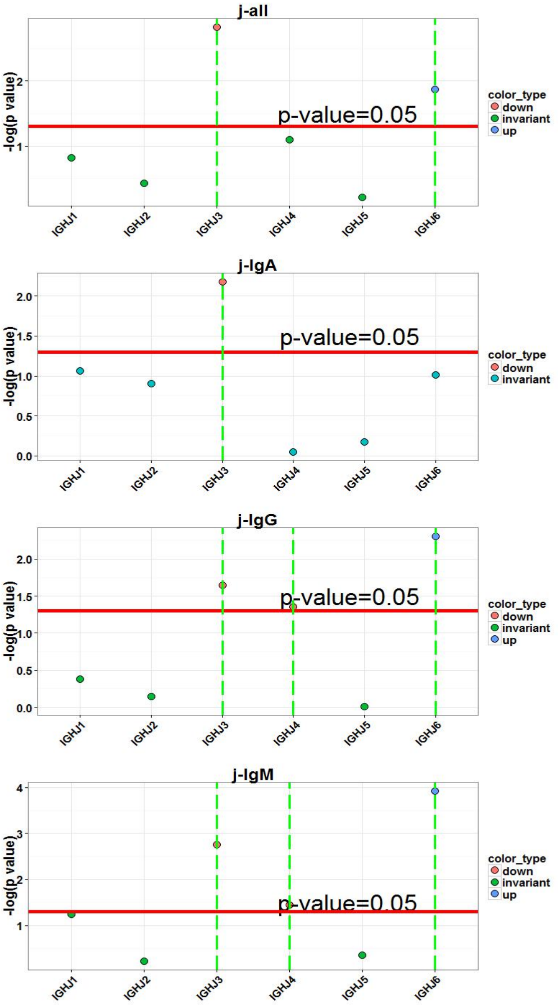
j-all
-log(p value)
p-value=0.05
color_type
down
invariant
up
IGHJ1
IGHJ2
IGHJ3
IGHJ4
IGHJ5
IGHJ6
j-IgA
j-IgG
j-IgM

(C)

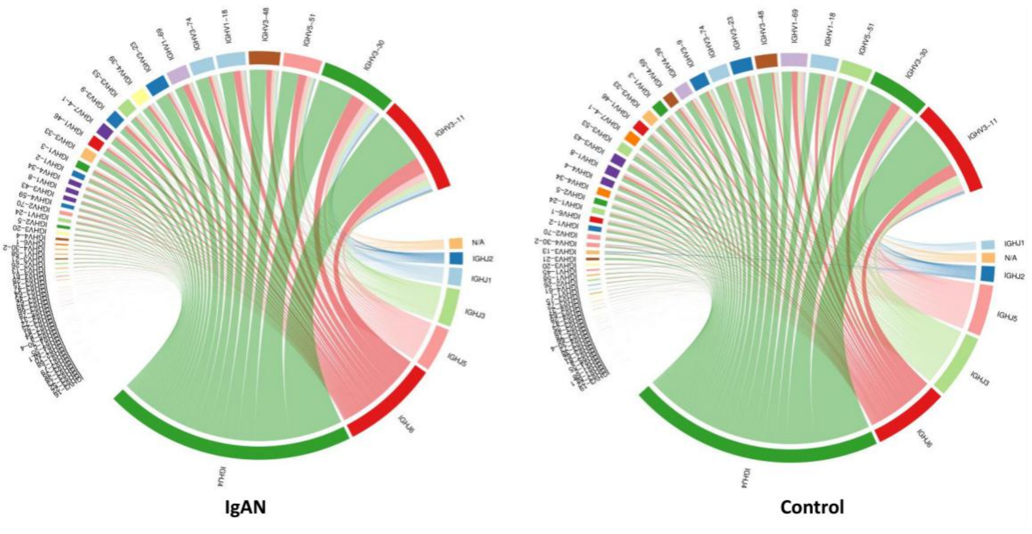
IgAN
Control

Fig. S3

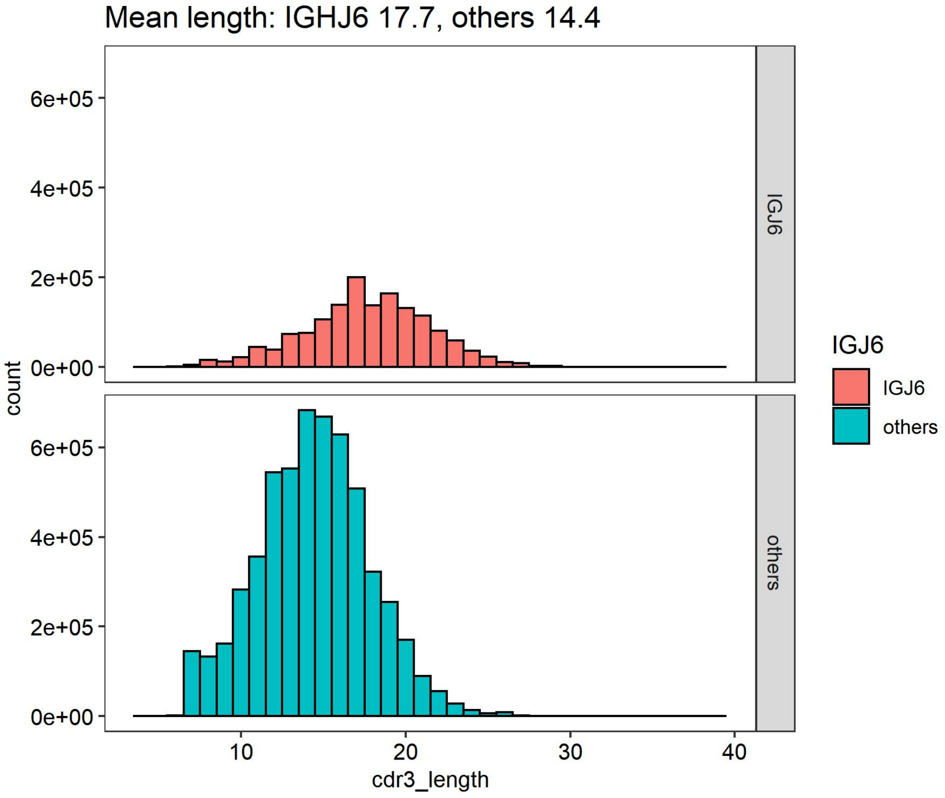

Fig. S4

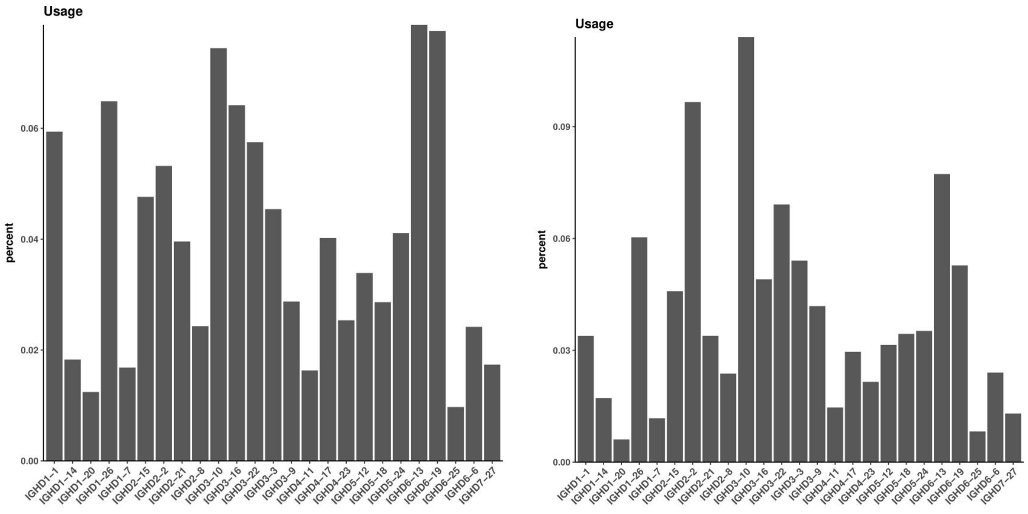

IgAN

NC

Fig S5

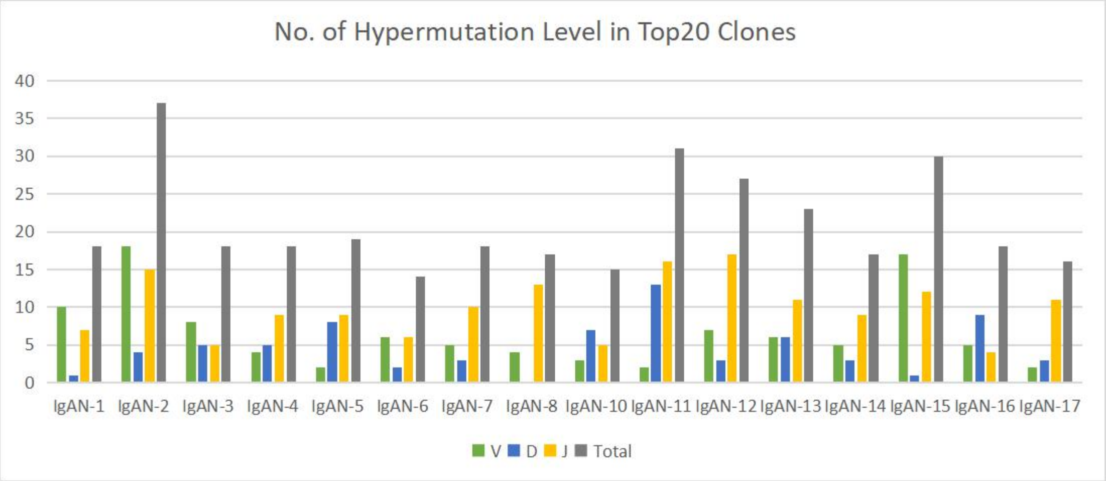

Fig. S6

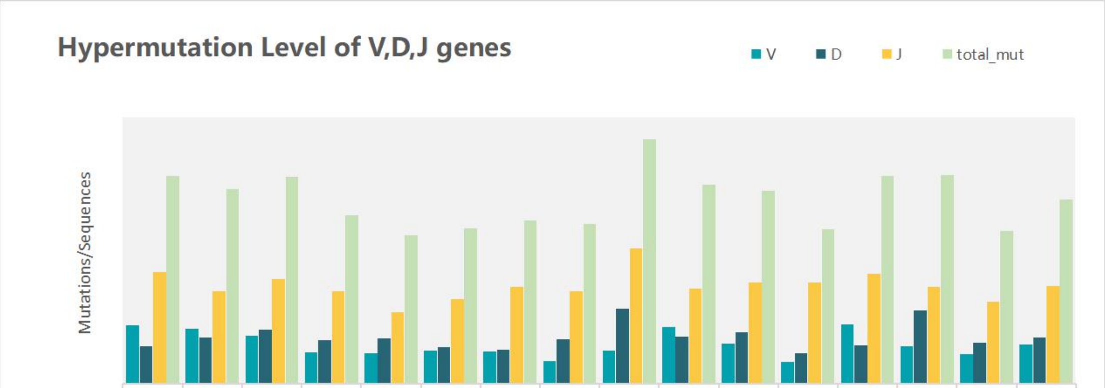

	IgAN-1	IgAN-2	IgAN-3	IgAN-4	IgAN-5	IgAN-6	IgAN-8	IgAN-10	IgAN-11	IgAN-12	IgAN-13	IgAN-14	IgAN-15	IgAN-16	IgAN-17	average
V	0.355	0.334	0.29	0.191	0.187	0.203	0.196	0.138	0.203	0.34	0.241	0.131	0.357	0.226	0.178	0.238
D	0.226	0.28	0.329	0.263	0.274	0.223	0.208	0.267	0.454	0.285	0.309	0.187	0.23	0.444	0.249	0.282
J	0.671	0.558	0.631	0.56	0.43	0.512	0.582	0.56	0.813	0.572	0.612	0.613	0.663	0.586	0.494	0.59
total_mut	1.252	1.172	1.249	1.014	0.892	0.938	0.986	0.965	1.47	1.198	1.162	0.931	1.25	1.256	0.921	1.11

Fig. S7

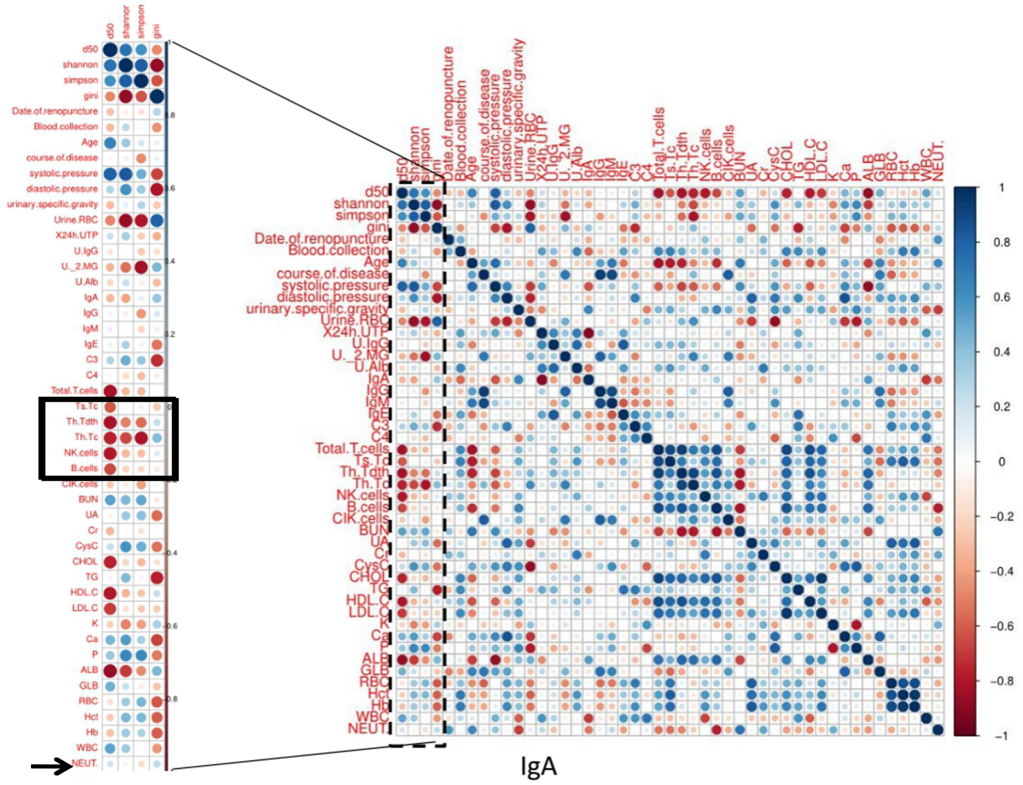

Supplement: Supplementary file 1 — Table S1. Clinical and biological data. A total of 13 patients with IgAN were recruited into the study (the information of seven normal candidates was not shown). Finally, eight IgAN samples and seven NC samples were qualified and later included in our statistical analysis. Table S2. Sequence statistics (reads per unique CDR3). Reads abundance in eight IgAN samples and seven NC samples. Table S3. Top 10 shared CDR3 amino acids sequence in IgAN and control. Table S4. The sequence information of PCR primers. Table S5. J‐gene usage. Fig. S1. Skewed CDR3 length distribution in patients with IgAN and controls. (A) CDR3 distribution and top 10 amino acids sequences in IgAN and NC. (B) Significance analysis of the length distribution between IgAN and control (*P < 0.05). Fig. S2. V and J gene usage in IgAN. (A) V and J gene usage of the variable region in IgAN and NC. (B) V gene usage preference in all antibody repertoire and IgA, IgM and IgG, respectively. (C) J gene usage preference in all antibody repertoire and IgA, IgM and IgG, respectively. Fig. S3. The average length of CDR3 with or without JH6. The average length of CDR3 is around 17.7, and the average length of CDR3 without JH6 is around 14.4. There are significant differences between JH6+ and JH6− with P < 0.001 (P = 2.2e−16). Fig. S4. D gene usage in IgAN. Fig. S5. Number of hypermutation level of top 20 clones in IgAN. Fig. S6. V, D and J gene mutation rate in IgAN. Fig. S7. Correlation of CDR3 diversity versus other clinical markers. Four statistical indices, including D50, Shannon, Simperson and Gini, show that several clinical markers are positively correlated with the CDR3 diversity (blue spots), whereas other markers are negatively correlated with the CDR3 diversity (red spots). [file FEB4-10-2702-s001.pdf]
